# ICU Length of Stay Patterns and In-Hospital Mortality: Clinical Determinants in a Tertiary-Care Hospital

**DOI:** 10.3390/healthcare14081092

**Published:** 2026-04-20

**Authors:** Carmen Pantis, Mihaela Simona Popoviciu, Timea Claudia Ghitea, Alina Manuela Pop, Roxana Daniela Brata

**Affiliations:** 1 Department of Medical Disciplines, Faculty of Medicine and Pharmacy, University of Oradea, 1 Decembrie, 410028 Oradea, Romania; carmen.pantis@didactic.uoradea.ro (C.P.); brata.roxanadaniela@didactic.uoradea.ro (R.D.B.); 2 Department of Preclinical Disciplines, Faculty of Medicine and Pharmacy, University of Oradea, 1 Decembrie, 410028 Oradea, Romania; alinatirb@uoradea.ro; 3 Department of Internal Medicine II, Diabetes Mellitus, Clinical County Emergency Hospital of Oradea, 410167 Oradea, Romania; 4 Pharmacy Department, Faculty of Medicine and Pharmacy, University of Oradea, 1 University Street, 410087 Oradea, Romania

**Keywords:** length of stay, intensive care units, mortality, critical illness, mechanical ventilation, renal replacement therapy, risk assessment

## Abstract

**Highlights:**

**What are the main findings?**

- ICU length of stay was not an independent predictor of mortality after adjustment.
- Organ support requirements were the strongest determinants of in-hospital death.

**What are the implications of the main findings?**

- Length of stay reflects disease severity rather than causality.
- Combining LOS with severity markers may improve risk stratification and ICU resource planning.

**Abstract:**

**Background**: Length of stay (LOS) reflects healthcare utilization but may also capture patient clinical trajectories. We investigated the relationship between LOS categories, organ support requirements, and in-hospital mortality. **Methods**: This retrospective observational study included 1332 consecutive adult ICU patients in a tertiary-care center. ICU LOS patterns were categorized using median-based and predefined cutoffs. Multivariable logistic regression was used to identify independent predictors of in-hospital mortality. **Results**: Prolonged ICU LOS was associated with higher crude mortality (61.0% vs. 43.5%, *p* < 0.001). However, in LOS-adjusted models, mortality was independently associated with mechanical ventilation (aOR 29.89, 95% CI 17.92–49.86), inotropic support (aOR 4.94, 95% CI 3.50–6.97), hemodialysis (aOR 5.43, 95% CI 2.52–11.72), older age, and diabetes mellitus. Prolonged LOS was not independently associated with mortality (aOR 0.93, *p* = 0.630). **Conclusions**: LOS reflects underlying disease severity rather than acting as an independent driver of mortality. Integrating LOS pattern assessment with markers of organ dysfunction may improve risk stratification and resource planning in hospitalized populations.

## 1. Introduction

Length of hospital stay is commonly used as a measure of healthcare utilization and system burden, but it also reflects the clinical course of ICU patients. In acute care settings, length of stay (LOS) often mirrors the evolution of disease severity, the occurrence of complications, and the response to treatment. Patients with rapidly resolving conditions typically experience shorter hospitalizations, whereas those with persistent organ dysfunction or complications frequently require prolonged care [1,2,3].

According to OECD and Eurostat data, hospital length of stay remains an important indicator of healthcare system performance, reflecting both disease severity and resource utilization across acute care settings. ICU-related hospitalization contributes substantially to bed occupancy, staffing demand, and healthcare costs, particularly in aging populations and patients with multi-organ dysfunction [4,5].

Although prolonged LOS is often associated with worse outcomes, clinically relevant endpoints include in-hospital mortality, need for prolonged mechanical ventilation, renal replacement therapy, discharge delay, and ICU readmission. Length of stay may represent not only disease severity but also clinical decision-making, discharge practices, and resource availability. Therefore, LOS should not be viewed solely as an administrative metric but as a potential indicator of patient trajectories during hospitalization [6,7,8].

Recent research has increasingly focused on trajectory-based approaches in acute and critical care, recognizing that patient outcomes are shaped by dynamic clinical evolution rather than static baseline characteristics. Trajectory analyses can capture patterns of deterioration, stabilization, or recovery over time and may provide additional prognostic insight beyond admission variables alone, including early deterioration trajectories, prolonged organ support trajectories, and delayed recovery patterns [9,10,11].

At the same time, organ dysfunction and the need for life-support interventions remain well-established drivers of mortality. It is unclear to what extent LOS independently contributes to outcome prediction when these severity markers are considered. Understanding the interplay between LOS categories, organ support requirements, and mortality may help refine risk stratification and resource planning [12,13,14].

Therefore, the present study aimed to investigate in-hospital mortality in relation to LOS patterns and clinical predictors in a real-world hospitalized cohort. Specifically, we sought to (1) compare characteristics of patients with short versus prolonged LOS, (2) identify clinical predictors of prolonged hospitalization, and (3) evaluate mortality predictors in models accounting for LOS stratification.

We hypothesized that prolonged LOS would be associated with higher in-hospital mortality; however, we further hypothesized that this association would be attenuated after adjustment for markers of organ dysfunction and life-support requirements, suggesting that LOS acts primarily as a proxy for disease severity rather than an independent determinant of outcome.

## 2. Materials and Methods

### 2.1. Study Design and Setting

This retrospective observational study was conducted at Bihor County Emergency Clinical Hospital, Romania, a tertiary-care referral center. The study included consecutive adult patients (≥18 years) admitted between January 2024 and December 2025, and this period did not overlap with the COVID-19 pandemic surge period.

The investigation focused on patients admitted to the intensive care unit (ICU), a mixed medical–surgical unit providing care for individuals with acute medical conditions, sepsis, respiratory failure, multi-organ dysfunction, and post-operative complications. The majority of admissions were medical rather than elective surgical cases. Admission was not restricted to a single index diagnosis; therefore, the cohort reflects a heterogeneous population of critically ill patients.

For contextual completeness, patients managed in medical wards during hospitalization were also recorded; however, length-of-stay analyses were based on ICU stay.

### 2.2. Study Population

All consecutive adult patients (≥18 years) admitted during the study period were screened for eligibility. Patients were included if complete data on ICU length of stay and in-hospital mortality were available.

Patients with missing key outcome variables (LOS or mortality) were excluded. No additional clinical exclusion criteria were applied to preserve real-world representativeness.

Missing covariate data were handled using complete case analysis. The proportion of missing data for individual variables was <5%.

**Figure 1:**
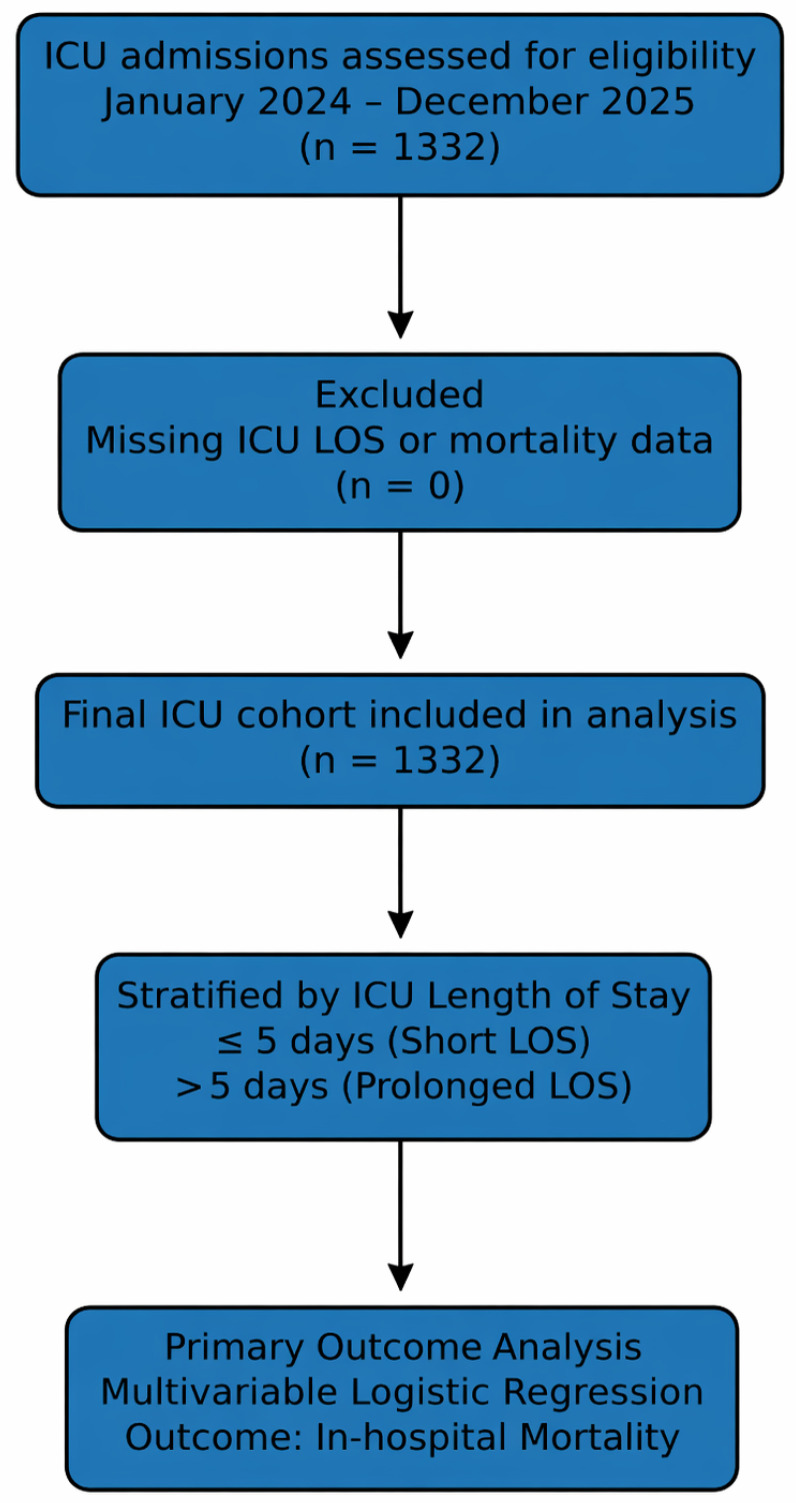
Flow chart.

All consecutive adult patients (≥18 years) admitted during the study period were screened for eligibility. The patient selection process is summarized in Figure 1.

### 2.3. Data Collection

Clinical and demographic data were extracted from electronic medical records. The following variables were collected:

- Age and sex;
- Comorbidities, including diabetes mellitus;
- Acute complications (sepsis, pneumonia, septic shock, acute respiratory failure, and acute renal failure);
- Organ support modalities (mechanical ventilation, inotropic support, and hemodialysis);
- ICU length of stay (days);
- In-hospital mortality.

All variables represented routinely documented clinical information.

Data were extracted from the hospital electronic medical record system by trained investigators. Diagnoses and complications were based on physician documentation and discharge summaries. Organ support variables were defined based on documented initiation of mechanical ventilation, vasoactive therapy, or renal replacement therapy during hospitalization. Acute complications were recorded if documented during hospitalization, regardless of whether they were present at admission or developed during ICU stay.

As data were obtained from routine clinical documentation, misclassification bias cannot be excluded. However, organ support interventions represent objective clinical events, which reduces measurement variability.

Comorbidities were recorded based on structured electronic medical record fields. Diabetes mellitus was consistently available and therefore included in the multivariable analysis. Other chronic conditions (e.g., heart failure, coronary artery disease, chronic pulmonary disease, malignancy) were variably documented and could not be reliably extracted for standardized modeling.

No self-reported variables were included.

### 2.4. Definitions and Trajectory Classification

Length-of-stay trajectories were defined using ICU LOS. For trajectory-based analyses:

- Short vs. prolonged LOS refers to ICU length of stay throughout the manuscript.
- Additional LOS categories (≤3 days, 4–7 days, and >7 days) were used to explore trajectory patterns.

Organ support was defined as the use of:

- Mechanical ventilation for respiratory failure.
- Inotropic/vasoactive support for circulatory failure.
- Hemodialysis for renal replacement therapy.

The median ICU LOS (5 days) was used as a pragmatic cutoff to define shorter versus prolonged ICU stay. Given the absence of universally accepted thresholds for prolonged ICU hospitalization and the right-skewed distribution of LOS data (range 1–162 days), the median provided a data-driven and distribution-sensitive method for dichotomization while preserving balanced group sizes for comparative analysis.

The LOS categories (≤3 days, 4–7 days, >7 days) were selected to reflect clinically interpretable short, intermediate, and prolonged ICU stays. While these thresholds are not formally validated cutoffs, they are commonly used descriptive intervals in ICU outcome studies and allow practical stratification of hospitalization patterns.

### 2.5. Outcomes

The primary outcome was in-hospital mortality.

Secondary outcomes included prolonged LOS (>median ICU LOS) and predefined LOS trajectory categories (≤3 days, 4–7 days, >7 days).

Analyses evaluating predictors of mortality were considered confirmatory, whereas LOS categories descriptive analyses were exploratory in nature. In-hospital mortality was defined as death occurring either in the ICU or after ICU discharge during the same hospitalization.

Comorbidities were defined based on documented chronic diagnoses recorded in the electronic medical record at admission. Due to database structure limitations, a formal comorbidity index (e.g., Charlson Comorbidity Index) could not be calculated.

### 2.6. Statistical Analysis

Continuous variables were expressed as mean ± standard deviation. Categorical variables were presented as counts and percentages.

Group comparisons according to LOS categories were performed using:

- Student’s *t*-test for continuous variables.
- Chi-square tests for categorical variables.

Multivariable logistic regression was conducted to identify predictors of mortality while adjusting for LOS. Results were reported as adjusted odds ratios (aOR) with 95% confidence intervals.

A two-sided *p*-value < 0.05 was considered statistically significant. Statistical analyses were performed using standard statistical software.

Given the number of variables tested, the multivariable model was restricted to clinically relevant covariates to reduce overfitting. The primary inference was based on the multivariable model evaluating mortality, which was prespecified according to clinical relevance.

Statistical analyses were performed using SPSS version 30 (IBM Corp., Armonk, NY, USA).

Univariable logistic regression analyses were additionally performed for all candidate predictors. Variables with *p* < 0.10 and clinically relevant covariates were subsequently entered into the multivariable logistic regression model.

Multivariable model covariates were selected based on clinical relevance and univariable association (*p* < 0.10).

Multicollinearity was assessed using variance inflation factors (VIF), with VIF > 5 considered indicative of collinearity.

Model fit was evaluated using the Hosmer–Lemeshow goodness-of-fit test.

### 2.7. Ethical Considerations

The study protocol was approved by the Ethics Committee of the University of Oradea (protocol code CEFMF/1, approved on 31 January 2023). The requirement for informed consent was formally waived by the same committee due to the retrospective design and anonymized data use.

## 3. Results

### 3.1. Demographic and Baseline Characteristics According to Length of Stay

A total of 1332 ICU patients were stratified according to ICU LOS into short (≤5 days) and prolonged (>5 days) groups.

Patients with prolonged ICU stays did not differ significantly in age, sex distribution, or diabetes prevalence compared to those with shorter stays. However, mortality was markedly higher among patients with prolonged LOS (61.0% vs. 43.5%, *p* < 0.001) (Table 1). The median ICU LOS was 5 days (IQR 3–9), which was used as the cutoff to define short versus prolonged ICU stay.

Additional baseline characteristics and ICU admission profile are provided in Table S1 (Supplementary Materials).

The distribution of ICU length of stay (LOS) in the study cohort (n = 1332) was as follows: median 5 days (interquartile range [IQR] 3–9), mean 7.69 ± 9.03 days, with a minimum of 1 day and a maximum of 162 days. The distribution was right-skewed, reflecting a subset of patients with markedly prolonged ICU stays.

### 3.2. Determinants of Prolonged ICU Length of Stay

Several acute complications and organ support interventions were associated with prolonged ICU length of stay (>5 days).

Acute respiratory failure was significantly more frequent among patients with prolonged LOS compared to those with shorter stays (75.7% vs. 54.8%, *p* < 0.001). Similarly, the need for mechanical ventilation was markedly higher in the prolonged LOS group (85.2% vs. 58.7%, *p* < 0.001).

Circulatory and renal support also showed significant associations. Inotropic support was more common in patients with prolonged LOS (36.7% vs. 25.8%, *p* < 0.001), as was hemodialysis (10.3% vs. 5.0%, *p* < 0.001).

In contrast, sepsis, septic shock, and acute renal failure alone did not show statistically significant differences between LOS groups.

Overall, interventions reflecting respiratory and multi-organ dysfunction were the strongest correlates of prolonged hospitalization (Table 2).

### 3.3. Predictors of Mortality in a LOS-Adjusted Model

A multivariable logistic regression model was performed to identify predictors of in-hospital mortality while adjusting for length-of-stay trajectory.

In univariable analysis, prolonged ICU LOS, acute respiratory failure, mechanical ventilation, inotropic support, and hemodialysis were all significantly associated with in-hospital mortality. However, after multivariable adjustment, prolonged LOS was no longer independently associated with mortality (aOR 0.93, 95% CI 0.69–1.25, *p* = 0.630), whereas organ support variables remained the strongest predictors of death (Table 3).

The multivariable model demonstrated adequate calibration (Hosmer–Lemeshow χ^2^ = 3.63, df = 8, *p* = 0.889). Multicollinearity diagnostics showed acceptable variance inflation factors for all included variables (VIF range 1.14–4.76), indicating no relevant multicollinearity.

**Figure 2:**
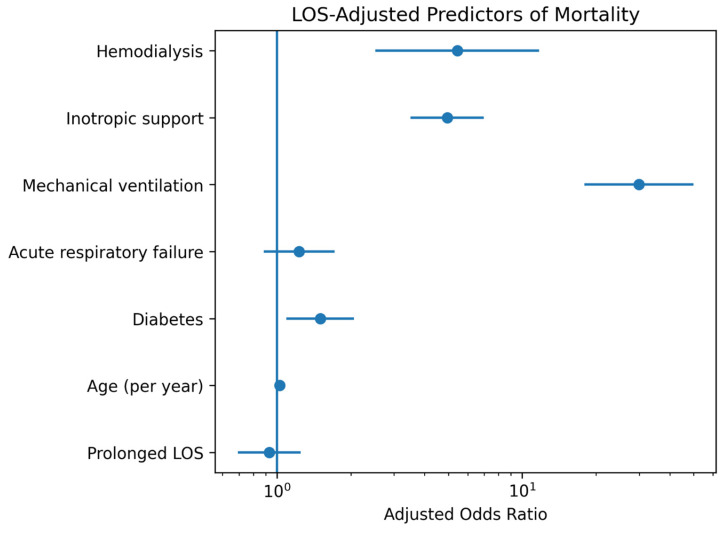
Forest plot of LOS-adjusted predictors of in-hospital mortality. Points represent adjusted odds ratios, and horizontal lines indicate 95% confidence intervals.

As shown in Figure 2, mortality was primarily driven by organ support requirements rather than length-of-stay duration itself.

### 3.4. Survival and Length-of-Stay Trajectories

A LOS categories analysis demonstrated a progressive increase in mortality across ICU length-of-stay categories.

Patients with short ICU stays (≤3 days) had the lowest mortality, whereas those with prolonged stays (>7 days) showed markedly higher mortality. This pattern suggests that extended hospitalization often reflects sustained critical illness and ongoing organ dysfunction.

Rather than LOS alone being causative, these trajectories likely capture the underlying severity and complexity of clinical evolution (Table 4). Mortality increased progressively across ICU LOS categories (≤3 days, 4–7 days, >7 days).

## 4. Discussion

This study examined in-hospital mortality and length-of-stay (LOS) trajectories in a large cohort of ICU patients, highlighting how clinical severity and organ support requirements shape both outcomes and hospitalization patterns. Several important observations emerge from our findings.

### 4.1. Length of Stay as a Reflection of Clinical Trajectory

Length of stay is often interpreted as a healthcare utilization metric, but it also reflects the clinical trajectory of ICU patients. In our cohort, prolonged ICU stays were associated with substantially higher mortality, suggesting that extended hospitalization frequently corresponds to sustained critical illness rather than recovery [1,15,16].

Patients with shorter stays exhibited lower mortality, which may reflect either milder disease or early stabilization. Conversely, prolonged stays likely capture patients experiencing persistent organ dysfunction, complications, or slow clinical resolution [17,18].

Importantly, LOS should not be viewed as a direct cause of mortality but rather as a marker of underlying disease complexity and trajectory. Studies evaluating emergency department LOS, particularly in trauma populations, have reported higher mortality among patients with shorter ED stays, likely reflecting early death among severely injured individuals. This phenomenon differs from ICU LOS, which reflects the duration of critical care rather than pre-admission stabilization time.

In the absence of APACHE II or SOFA scores, organ support variables were used as pragmatic surrogate markers of baseline and evolving severity.

### 4.2. Organ Dysfunction as the Primary Driver of Outcomes

When adjusting for LOS, mortality was primarily driven by markers of organ dysfunction and life-support requirements. Mechanical ventilation, inotropic support, and hemodialysis remained the strongest predictors of death [19,20,21]. The high adjusted odds ratio observed for mechanical ventilation reflects the strong association between invasive respiratory support and critical illness severity rather than statistical instability.

These findings reinforce the concept that physiological severity outweighs hospitalization duration in determining prognosis. LOS alone may therefore be insufficient for risk stratification without considering the clinical context and organ support needs [22,23,24,25,26].

While the association between organ dysfunction and prolonged hospitalization is clinically intuitive, our findings clarify that LOS itself does not independently drive mortality once severity markers are considered. This distinction is important for interpreting LOS as a quality indicator in health systems.

### 4.3. LOS Stratification

The trajectory analysis demonstrated a stepwise increase in mortality across LOS categories. This pattern supports the idea that patient evolution over time provides prognostic information beyond static admission characteristics [27,28,29].

LOS pattern frameworks are increasingly recognized in acute care research, as they capture dynamic disease progression. Our results suggest that combining LOS patterns with organ support data may offer a pragmatic approach to monitoring severity [30,31,32].

### 4.4. Clinical Implications

The independent predictors identified in our model—advanced age, diabetes mellitus, and the need for organ support—are largely non-modifiable at the time of ICU recognition and should therefore be interpreted primarily as prognostic markers rather than direct therapeutic targets. In particular, mechanical ventilation, vasoactive support, and hemodialysis reflect the severity of physiological derangement rather than modifiable causal risk factors. Consequently, the principal utility of the model lies in prognostication, early risk stratification, and ICU resource allocation, including bed occupancy, ventilatory equipment, dialysis support, nursing workload, and physician coverage, rather than direct mortality reduction strategies [33,34,35].

### 4.5. Strengths and Limitations

This study benefits from a large real-world ICU cohort and routinely collected clinical data, enhancing generalizability and providing a broad view of hospitalization trajectories.

Formal severity scores such as APACHE II or SOFA were not available in the electronic database. Organ support requirements were therefore used as pragmatic markers of acute severity.

However, several limitations should be acknowledged. The retrospective design limits causal inference, and unmeasured confounding cannot be excluded. Formal severity scores were not available, and LOS categories were based on cohort-specific distributions. Additionally, single-center data may limit external validity.

Important laboratory parameters such as complete blood count, renal function tests, electrolytes, serum glucose, albumin, and lactate were not consistently available in a standardized format across the entire cohort and therefore were not included in the multivariable analysis. Inclusion of worst values during ICU stay may also have introduced time-dependent bias relative to organ support initiation. The absence of a comprehensive comorbidity index (e.g., Charlson Comorbidity Index) represents a limitation and may have resulted in residual confounding.

Surgical intervention status was not consistently recorded in a structured format and therefore could not be included in the multivariable model.

LOS is inherently influenced by survival time. Early death necessarily results in shorter LOS, introducing potential reverse causation and time-dependent bias. Therefore, LOS should be interpreted as a marker of clinical evolution rather than a causal determinant of mortality. Only ICU LOS was analyzed. Total hospital LOS, including post-ICU ward stay, was not available in a standardized format.

### 4.6. Future Directions

Future research should incorporate prospective designs and dynamic severity scoring to better characterize trajectory patterns. Evaluating temporal changes in organ support requirements may further refine prognostic models.

Future studies should also investigate the impact of staffing models, including nurse-to-patient ratios and physician coverage, on prolonged ICU length of stay and patient outcomes.

Overall, our findings indicate that LOS categories reflect underlying disease severity rather than acting as independent drivers of mortality. Organ dysfunction and life-support requirements remain the dominant determinants of outcome. Integrating LOS patterns assessment with clinical severity markers may enhance risk stratification in ICU patients.

## 5. Conclusions

Length-of-stay trajectories in ICU patients reflect the underlying course and severity of illness rather than acting as independent determinants of mortality. Prolonged ICU stays were associated with higher mortality, but this relationship was largely explained by the presence of organ dysfunction and the need for life-support interventions.

Organ support requirements remained the strongest predictors of death even after accounting for LOS, underscoring the dominant role of physiological severity in shaping outcomes. LOS category assessment may nevertheless provide useful contextual information for monitoring patient evolution and anticipating resource needs.

Integrating LOS stratification with clinical severity markers could support more nuanced risk stratification and care planning. Future prospective studies should explore dynamic models that incorporate temporal changes in organ dysfunction to better characterize patient trajectories and outcomes.

## Figures and Tables

**Table 1:** Baseline demographic and comorbidity characteristics according to ICU length of stay.

Variable	Short LOS (≤5 Days) (n = 722)	Prolonged LOS (>5 Days) (n = 610)	*p*-Value
Age, mean ± SD (years)	68.1 ± 13.0	67.8 ± 13.2	0.663
Male sex, n (%)	350 (48.5)	321 (52.6)	0.146
BMI, mean ± SD (kg/m^2^)	31.6 ± 11.58	35.2 ± 17.12	0.002
Diabetes mellitus, n (%)	227 (31.4)	205 (33.6)	0.434
ICU LOS, median (IQR), days	3 (2–4)	8 (6–13)	<0.001
In-hospital mortality, n (%)	314 (43.5)	372 (61.0)	<0.001
Hypertension, n (%)	628 (86.9)	574 (94.0)	0.027
Smoking, n (%)	274 (37.9)	189 (30.9)	0.042

Data are presented as mean ± standard deviation (SD) or number (percentage), as appropriate. Comparisons between groups were performed using Student’s *t*-test for continuous variables and chi-square tests for categorical variables.

**Table 2:** Clinical Predictors of Prolonged Length of Stay.

Variable	Short LOS (≤5 Days) (n = 722)	Prolonged LOS (>5 Days) (n = 610)	*p*-Value
Sepsis, n (%)	113 (15.7)	119 (19.5)	0.076
Septic shock, n (%)	61 (8.4)	61 (10.0)	0.377
Acute respiratory failure, n (%)	396 (54.8)	462 (75.7)	<0.001
Acute renal failure, n (%)	176 (24.4)	170 (27.9)	0.166
Mechanical ventilation, n (%)	424 (58.7)	520 (85.2)	<0.001
Inotropic support, n (%)	186 (25.8)	224 (36.7)	<0.001
Hemodialysis, n (%)	36 (5.0)	63 (10.3)	<0.001

Values are presented as percentages (%). *p*-values were calculated using chi-square tests for comparisons between groups. LOS, length of stay.

**Table 3:** Univariable and multivariable predictors of in-hospital mortality.

Predictor	Crude OR	95% CI	*p*-Value	Adjusted OR	95% CI	*p*-Value
Prolonged LOS (>5 days)	2.03	1.63–2.53	<0.001	0.93	0.69–1.25	0.630
Age (per year)	-	-	-	1.03	1.01–1.04	<0.001
Diabetes mellitus	1.11	0.89–1.39 *	0.434	1.50	1.09–2.06	0.012
Acute respiratory failure	2.57	2.03–3.25 *	<0.001	1.23	0.88–1.72	0.221
Mechanical ventilation	4.05	3.17–5.17 *	<0.001	29.89	17.92–49.86	<0.001
Inotropic support	1.67	1.31–2.13 *	<0.001	4.94	3.50–6.97	<0.001
Hemodialysis	2.19	1.42–3.39 *	<0.001	5.43	2.52–11.72	<0.001

Adjusted odds ratios (ORs) and 95% confidence intervals (CIs) were obtained from multivariable logistic regression analysis. *p*-values < 0.05 were considered statistically significant. LOS, length of stay; OR, odds ratio; CI, confidence interval, * Univariable OR estimates derived from binary contingency analyses and confirmed by logistic regression screening.

**Table 4:** Mortality Across LOS Stratification.

ICU LOS Category	n	Mortality (%)
≤3 days	457	42.0
4–7 days	451	49.9
>7 days	422	63.7

Values are presented as counts (n) and percentages (%). Mortality represents in-hospital mortality within each ICU length-of-stay category. ICU, intensive care unit; LOS, length of stay.

## Data Availability

The data presented in this study are available on request from the corresponding author. The data are not publicly available due to privacy or ethical restrictions.

## Supplementary Materials

The following supporting information can be downloaded at: https://www.mdpi.com/article/10.3390/healthcare14081092/s1, Table S1. ICU admission profile and baseline severity indicators of the study cohort (n = 1332).

## Abbreviations

The following abbreviations are used in this manuscript:

LOS	Length of Stay
ICU	Intensive Care Unit
SD	Standard Deviation
OR	Odds Ratio
aOR	Adjusted Odds Ratio
CI	Confidence Interval
ARF	Acute Respiratory Failure
