# Supplementary material for: ICU Length of Stay Patterns and In-Hospital Mortality: Clinical Determinants in a Tertiary-Care Hospital
Source: Healthcare (Basel). 2026 Apr 20;14(8):1092. doi: 10.3390/healthcare14081092 (PMC13115808; doi:10.3390/healthcare14081092)
Supplement: Supplementary file 1 [file healthcare-14-01092-s001.zip › healthcare-4207723-supplementary.pdf]

**Supplementary Table S1**. ICU admission profile and baseline severity indicators of the study cohort (n = 1,332).

| **Variable** | **n (%)** |
| --- | --- |
| Mixed medical ICU admissions | 1,332 (100) |
| Sepsis | 232 (17.4) |
| Septic shock | 122 (9.2) |
| Acute respiratory failure | 858 (64.4) |
| Acute renal failure | 346 (26.0) |
| Mechanical ventilation required | 944 (70.9) |
| Inotropic support required | 410 (30.8) |
| Hemodialysis required | 99 (7.4) |

Values represent the prevalence of major admission-related diagnoses and severity indicators documented during ICU stay. Variables are not mutually exclusive because patients may present with multiple concurrent conditions and organ support requirements.
